# A Biological Stone from a Medieval Cemetery in Poland

**DOI:** 10.1371/journal.pone.0109096

**Published:** 2014-10-02

**Authors:** Judyta J. Gładykowska-Rzeczycka, Dariusz Nowakowski

**Affiliations:** 1 Professor retired, Marusarzówny, Gdańsk, Poland; 2 Department of Anthropology, Wrocław University of Environmental and Life Sciences, Kożuchowska, Wrocław, Poland; University of Kansas, United States of America

## Abstract

A review of the literature shows that origination of biological stones as well as their pathogenesis mostly depend on the environmental factors. As a result, the structural spectrum of such calculi and their chemical composition are highly diversified. It is well known that biological stones are formed mostly in the digestive and urinary tracts. However, it has been demonstrated that this kind of stony structure can be also, though rarely, found in circulatory and reproductive systems, skin, mucosa, and tear ducts. Although in palaeopathology, the list of biological stones is enriched by stony tumours and/or discharges, it is very difficult to uncover the small size deposits in excavation material. In the literature such findings, originating from different countries and centuries, are few. The described stone was found among the bones of an adult individual in the medieval cemetery of Gdańsk (Poland). The SEM, X-ray spectrometer and chemical evaluation revealed that it was a bladder calculus.

## Introduction

The origin of biological stones and, what is most important, their pathogenesis overall depend on the environment and individual predispositions [1]. As a result, the structural spectrum of such calculi and their chemical composition are highly diversified [2]–[4]. Biological stones are formed mostly in the digestive and urinary systems, less often in vascular system, lacrimal glands, mucosa, tendons, cartilage, or skin [5]–[6].

Although in palaeopathology stony neoplastic tumours [7]–[8] and/or exudates [9] are found, it is very difficult to uncover the small size deposits in excavated skeletal material (except for mummies); such findings, originating from different countries and centuries, are few [3], [10]–[11].

Besides the calcified echinococcus cyst from an early mediaeval cemetery in Dziekanowice [4], [12], this case report is the first describing a fossil biological stone from Poland.

The aim of our research was to: identify the type of organic stone, as well as its physical and chemical structure, ascertain the probable causes of its formation, determine the individual's sex and age, and identify any other evidence of pathological change, expanding the existing knowledge of the socio-economic conditions in mediaeval Gdańsk where it was found and add to the existing database of palaeopathological examples of organic stone discoveries.

Studies of human burials on all continents are a valuable source of information about the conditions of life in mediaeval and prehistoric populations. Archaeological and anthropological analysis provides valuable information about the socio-economic status of a population. Besides human and often animal bones, burials often contain archaeological artefacts [13]. However, among the remains it is sometimes possible to find other objects, such as calcified or ossified masses and calculi accompanying the skeleton [14]. These objects often include urinary or renal calculi, gallstones, leiomyomas, ovary cysts and calcified ovaries. The presence of these objects among skeletal remains has been mentioned in publications on burials from different parts of the world [4], [12]–[13], [15]–[25]. Most of the previously discovered stones were collected from mummies [16], [23]. Publications on such objects from the territory of Poland are scarce, except the case of the mummy from Wrocław described by Borysławski [26]. Besides the calcified echinococcus cyst from an early mediaeval cemetery in Dziekanowice [4], [12], this is the first case of a fossil biological stone in Poland. Many researchers indicate the relationship of the presence of biological stones to climate. In their opinion the occurrence of this pathology is associated with environmental factors such as hot and dry climate, combined with a low-protein diet. A cereal-based diet also has a significant impact on the formation of biological stones [14]. It is believed that urolithiasis, and the associated presence of urinary stone, is common in industrial societies [27]–[28]. In contrast, gallstones have genetic causes and are age-dependent [29]. It is also noteworthy that the formation of biological stones and, what is most important, their pathogenesis, depend on the overall environment and individual predispositions [1]. Palaeopathological studies of these cases may provide valuable information about the diseases of the past. However, some adverse factors, such as taphonomic conditions or careless excavations may hinder attempts to discover, document, analyse and identify these objects [18], [22], [30]. Furthermore, the diagnosis of biological stones requires a very complicated procedure [10]–[11], [18], [22]. Several techniques are used to diagnose these rare historical and ancient objects. Generally, conventional techniques used in the diagnosis include morphological, radiographic and microscopic analysis [18], [21]–[22], [24]. Microscopic and chemical analysis of sections reveal information about the internal matrix [24], [31]. Also other techniques, including X-ray diffraction (XRD), scanning electron microscope (SEM), micro chemical analysis (EDS), X-ray fluorescence (XRF), are used [13], [17]–[16], [19]–[21], [23]–[24], [32]. Because the biological stones are the result of various interrelated developments, all these tests allow to obtain information on the mineral composition and formation of these pathologies in the body.

## Materials and Methods

**Figure 1:**
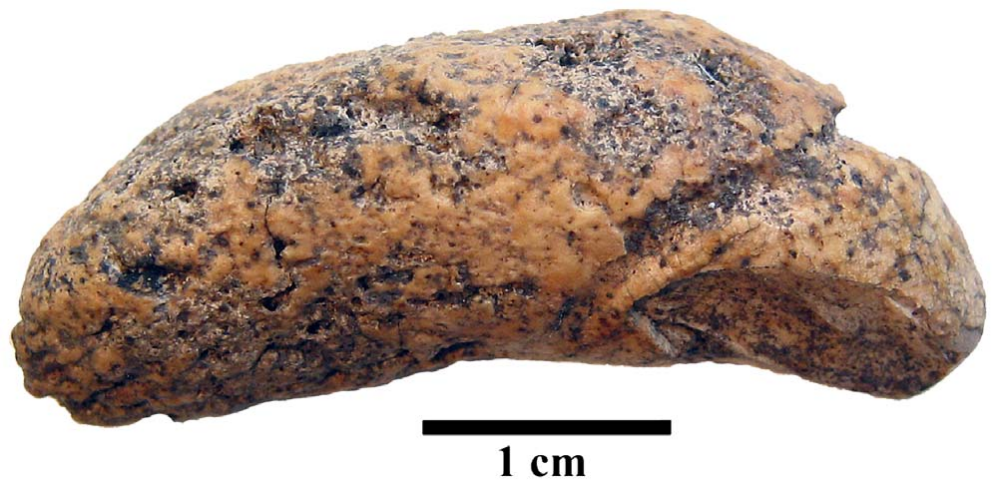
A macroscopic view of the stone.

The material comes from Gdańsk situated on the Baltic coast in the northern part of Poland. In 2001 archaeological excavations conducted by the Gdańsk Archaeological Museum at the Market Hall in Gdańsk's Old Town brought to light the remains of a settlement, the foundations of a church and an extensive, multilevel burial ground representing the largest ever mediaeval cemetery discovered in Gdańsk. The 10th–13th-century market settlement noted at this site featured a cemetery at its western end and was situated on a major trade route referred to in written records as the *via mercatorum*, i.e. "merchants road". Archaeological investigations revealed that the burial ground had been in use from the latter half of the 10th century right up until 1813. In total, the cemetery, which had remained in use for at least 800 years, yielded around 1000 graves in various states of preservation. As the graves occupied several levels, successive burials often damaged or disturbed the earlier ones. The construction and expansion of the Romanesque church, followed by the erection of the Dominican monastery and later the Market Hall all wrought significant damage on the burial ground. In consequence, approximately 30% of the skeletal material was very badly damaged. Some of the secondary burials contained several disarticulated skeletons which were recorded as concentrations. It was in one of these (No. 300), among the remains of four incomplete skeletons, that the stone discussed herein was found (Fig. 1). The date determined for this concentration was fairly broad: mid-10th to mid-14th century [33]. The research material are in repository in the Gdańsk Archaeological Museum, ul. Marjacka 27/28, Gdańsk, Poland.

Standard anthropological methods were used to determine the age and sex of skeletons from the concentration of burial No. 300.

**Figure 2:**
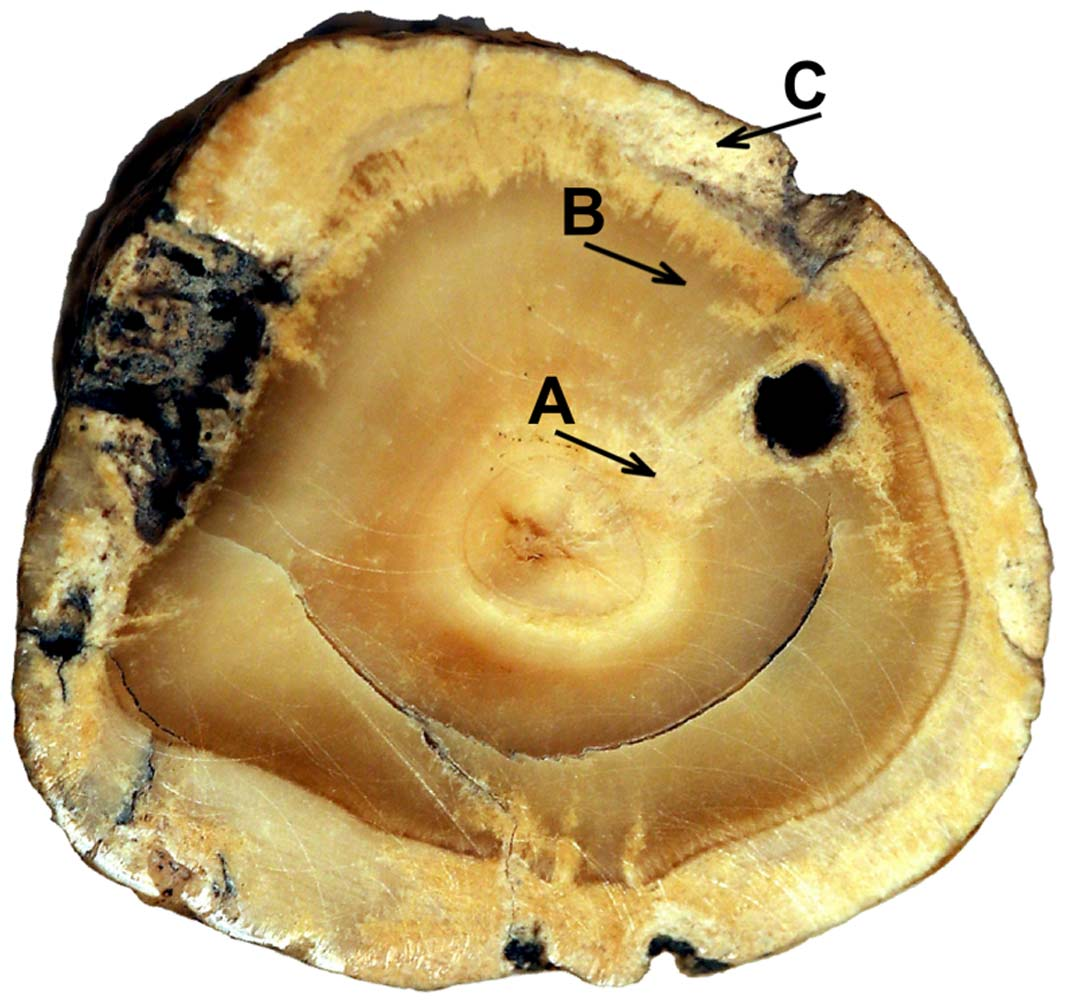
Localization of chemical analysis points on transverse section of the stone (description in the text). Concentric layers of the stone.

**Figure 3:**
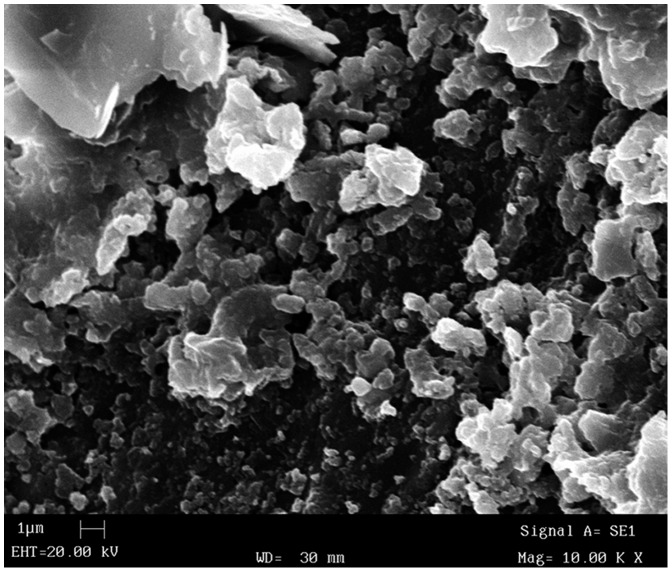
Localization of chemical analysis points on transversal section of the stone.

**Figure 4:**
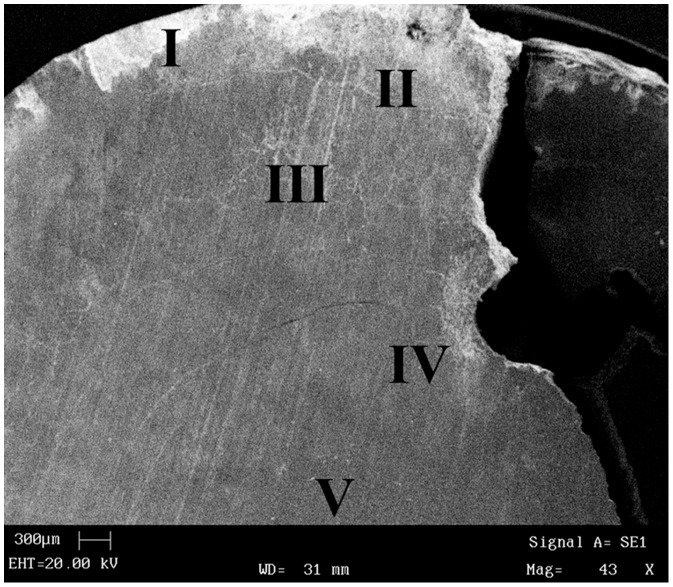
Scanning electron micrograph of crystallic structure of the stone.

In order to examine the shape, structure and chemical composition of the stone the following methods were employed: 1) macroscopic evaluation: the length, width, and thickness were measured by geometric compass while the circumference was measured by using a tape (±0.5 mm). The weight was determined by electronic balance (±0.01 g). Stone sections were cut using diamond saw blades (0.2 mm thick), and the cut surfaces were polished using diamond abrasive wheels (180, 600 and 1200 mesh). Sections (Fig. 2) were analysed and photographed with scanning microscopy, SEM, LEO 435 VP with magnifications ×43 (Fig. 3) and ×1000 (Fig. 4). 2) Chemical evaluation: Composition of chemical elements was determined using X-ray spectrometer "Rontec GmbH" at five different points of the cut surface (transverse section) of the analysed stone (Fig. 3).

The most important chemical compounds of calcium, ammonium, magnesium, uric acid, oxalate, phosphate, and cystine were determined with reagent kit Ecoline Urinary calcium analysis (DiaSys Diagnostic Systems GmbH). A comparison between Ecoline Urinary calcium analysis and infrared spectrometry and X-ray examination shows that the methods used give almost identical results [34]. The composition of three layers was analysed (inner layer with a core, middle, and external).

## Results

Although the bones from assemblage No. 300 are not well preserved, it was possible to establish that these skeletal remains represented four individuals: an *adultus* (20–39 years old) female, a *maturus* (40–55 year-old) male, one teenager, and one foetus. It is possible that the biological stone belonged to one of the individuals (except the foetus) found in this assemblage. The stone is kidney-shaped and light brown in colour with a few darker spots and a rough, porous surface. The size is about 39.0 mm × 14.0 mm × 12.5 mm (length, width and thickness, respectively) and the circumference is 44 mm. The weight is 6.87 g. On the cut surface of transverse section, approximately at half of its length there are clear, yellowish, concentric layers around the inner core (Fig. 2).

The percentage composition of the elements determined in the studied five points of the stone varies (Table 1).

**Table 1:** Percentage composition of the elements in points I–V (according to Figure 3).

Elements	point I	point II	point III	point IV	pointVI
C	8.62	10.72	9.56	11.18	21.34
O	45.10	40.47	34.46	42.65	44.06
Na	0.37	0.86	0.41	0.34	0.58
P	13.32	15.48	16.70	14.89	11.79
K	0.31	–	–	–	0.53
Ca	29.75	28.43	36.10	29.60	17.70
S	0.36	0.83	0.77	0.82	0.99
Mg	0.06	0.32	0.22	0.52	0.33
Cl	0.39	1.44	1.69	–	0.63
Si	1.28	–	–	–	1.38
Al	0.44	1.46	–	–	0.67

The chemical composition is as follows: calcium carbonate [CaCO_3_], calcium phosphate [Ca_3_(PO_4_)_2_], calcium oxalate [CaC_2_O_4_].

Compounds such as ammonium urate [NH_4_·C_5_H_4_N_4_O_3_], uric acid [C_5_H_4_N_4_O_3_], cystine [C_6_H_12_N_2_O_4_S_2_] were not determined. In the inner layer (Fig. 2, arrow A) the following compounds were determined: calcium phosphate (70%), calcium carbonate (25%) and calcium oxalate (5%). The analysis of the middle layer (Fig. 2, arrow B) revealed the presence of calcium phosphate (60%), calcium carbonate (15%), calcium oxalate (0%), while the external layer (Fig. 2, arrow C) consisted mostly of calcium phosphate (65%), calcium carbonate (20%), calcium oxalate (10%). In the SEM analysis a crystalline structure of the stone is visible (Fig. 4).

## Discussion

The gross morphology, size, laminar structure, SEM analysis and chemical composition of the analysed stone indicate that its core is made of homogenous crystalline structure, which gradually, through the process of stratification, became a consistent spherical concretion.

Diagnosing the causes of the stone formation should be made by differentiation. Since the skeletons found near the stone showed no pathology, it was concluded that the stone was not a result of ossification of the tissues. Therefore, one can assume that the origin of the biogenic object was either a tissue calcification or a calculus formation [18], [24]. According to Armentano et al. [25], clinical studies suggest some conditions of such calcifications with respect to morphological appearance: benign tumors, neoplasms, infections, vascular calcifications and other causes. No traces of tuberculosis were observed in the skeletons, therefore tuberculoma calcification was excluded [35]. On the surface of the stone there is no trace of blood vessels, so it is not a calcified ovary, ovarian cyst, lithopaedion or a calcified lymph node [21]. In contrast, the object has a characteristic chemical composition. It shows that it is a bladder stone, which Abboud [36] defined as hard masses comprised of organic compounds and inorganic crystals, that are harvested crystals in the kidney or urinary ducts. The diagnosis of bladder stone as also confirmed by the concentric arrangement of the layers which is typical of vesicular calculi [19]–[20].

Urinary stones vary in size from a few millimetres to a few centimetres [36]. Stones with a large diameter are classified as "giant". For example, Giufra et al. [23] classified a bladder stone of a 7.5 cm diameter found in the pelvis of a mummy as a giant bladder stone. Therefore, the object described here can be considered large.

The composition of urinary system stones allows for the determination of the type of stones. The most common stones are those built of either calcium oxalate or phosphate [37]–[39]. The chemical composition of particular layers is not uniform, which indicates inconsistent chemical environment during the stone growth. Detailed chemical analysis revealed the presence of significant amounts of phosphate compounds throughout the layers. According to medical [38] and palaeopathology literature [10]–[11], [19]–[20] it is problematic to determine what causes the process of phosphate compound accumulation in the body. D'Alessio et al. [40] argue that urinary stones predominantly composed of calcium phosphate develop in alkaline or weakly acidic urine. However, it has been recorded that such process can be initiated as a result of elevated (above 8) pH and during inflammation attenuated by anaerobic bacteria. High content of phosphate compounds suggests that the diet in this case was rich in phosphate compounds [39], for example in fish meat. Other animal proteins were present in trace amounts, or not at all. Consistent presence of oxalate compounds in both internal and external layers indicates that the diet, at the beginning and the end of the calculus growth, was rich in highly acidic food (e.g., sorrel plant). On the other hand, complementary EDS analysis suggested that the basic structure consisted of calcium and phosphate (Table 1). The analysis did not demonstrate a sharp differentiation of Ca in the whole cross-section of the stone from the core to the cortex, and increased value of phosphorus was present in analyses of II-IV (Fig. 3). This confirms the observations of Robertson [41] that urinary stones are often present in societies whose diet is rich in plant products and low in meat and dairy products. It is quite possible that each phase of the stone growth was affected by negative health condition, including prolonged starvation, or other diseases, which resulted in production of alkaline urine. This condition most likely led to ascending infection to the kidneys and/or hydronephrosis with uremia [42]. A rough, porous surface indicates a very aggressive bacterial inflammation developed at the final stage of stone formation, which could be a major cause of death.

## Conclusion

The chemical analysis of the stone, combined with anthropological methods, macroscopic evaluation and SEM, has enabled several conclusions.

Its composition demonstrates that it is a bladder stone and that the diet of the affected individual consisted of highly acidic food. Evidence of inflammatory processes resulted from a chronic disease which was probably the cause of death.

At this stage of research into the burial site (multidisciplinary analyses are currently in progress) it is too early to reach any more detailed conclusions relating to diet, or the socio-economic conditions of the population represented. We can only speculate that this individual was not one of Gdańsk's affluent citizens, who doubtless took advantage of the stone removal procedures already available at the time.

In addition to preliminary reports [43]–[44], this is the first recorded instance in Poland of a biological stone noted in a skeleton recovered from an excavation.
